# The show must go on: fostering residents’ sustainable employability in medical education – a qualitative exploration of the Resident Leadership Program

**DOI:** 10.1186/s12909-024-06053-2

**Published:** 2024-10-22

**Authors:** Iris van de Voort, Ian Leistikow, Jan-Willem Weenink

**Affiliations:** 1 Erasmus School of Health Policy and Management, Erasmus University Rotterdam, Oudlaan 50, Rotterdam, 3062 PA The Netherlands; 2 Dutch Health & Youth Care Inspectorate, Ministry of Health, Welfare & Sport, Utrecht, The Netherlands

**Keywords:** Sustainable employability, Residents, Self-regulation, Context of medicine, Leadership development, Quality improvement work

## Abstract

**Background:**

Residents’ sustainable employability (SE) is threatened by high burn-out rates, sleep deficits, and career dissatisfaction. Medical education may contribute to residents’ SE by providing them with opportunities to influence their employment contexts and to develop conscious self-regulation. This paper explores how residents, participating in the Resident Leadership Program (RLP), are enabled to work on, and learn about, their SE.

**Methods:**

The RLP took place between February and July 2021 and consisted of lectures on, and practice in, quality improvement (QI) work. SE was the theme that governed residents’ QI projects. In this study, residents were interviewed individually before the program (*n* = 8), were observed while participating in the program (45 h) and were interviewed in three groups after the program (*n* = 8). The data were analysed in accordance with the ‘flexible-coding’ method.

**Results:**

The findings are presented in four ‘acts’ mirroring an unfolding play as a metaphor to show how residents’ understanding of context, self-regulations, and quality improvement work—relevant to their SE—changed in the RLP. The acts include *‘setting the stage’*, describing how residents experienced the context of medicine; *‘acting the part’*, depicting how residents managed their employment contexts using self-regulation; ‘*changing the décor*’, elaborating on residents’ QI projects; and *‘growing one’s role’*, presenting residents’ take-aways from participating in the RLP that may benefit their SE. These take-aways encompassed awareness of the importance of SE, a reconsideration and/or adjustment of self-regulation, feeling better equipped to navigate employment contexts, and increased joy in work because of contributing to peers’ SE through QI work.

**Conclusions:**

Our results indicate that medical education is a fruitful environment for providing important lessons and tools for residents to work on and learn about their SE, likely benefiting their SE throughout their careers.

## Background

The sustainable employability (SE) of medical residents may well be under threat. SE can be understood as an individual’s ability “to function as effectively, efficiently and healthily as possible within a given (un)employment context, now and in the future” [1]. Individuals’ SE is a social construct that can be meaningfully captured through indicators spanning three domains [2]: wellbeing, health, and competence. For many residents, each of these domains is increasingly compromised, and as such, so may their SE. Well-being is affected by impaired empathy, career regret and/or dissatisfaction [3, 4]. Health is impaired through high burn-out rates and sleep deficits [5, 6]. Competence (development) is not always perceived as adequate with residents feeling unprepared for independent practice, especially in terms of non-clinical tasks, because of unmet educational needs and reduced clinical encounters [7–10].

Diminished residents’ SE also affects quality of care. Indicators or ‘building blocks’ of residents’ SE, such as perceived health status (e.g., burnout or depression) or fatigue, are associated to medical errors, suboptimal care, and lower provider-perceived quality of care [11–13]. In addition, compromised SE may also result in sickness absence, turnover, and a decreased attractiveness of pursuing a career in medicine, further depleting already scarce personnel resources [2, 14]. It follows that sustaining residents’ employability is imperative for sustaining health system functioning [15]. However, academic research has not yet explored the SE of residents.

Compromised SE may be “an inherent – and potentially unavoidable – risk (…) [of] a career in medicine” [16]. Learning and practicing medicine is demanding, occurs in suboptimal and inefficient environments, and involves much uncertainty [16]. Residents are also the most frequent ‘target’ of mistreatment by colleagues [17] and generally experience work-home interference and poor social support in the workplace [18, 19]. Thus, the context of medicine plays an important yet often debilitating role in residents’ SE [20, 21]. Research, however, has shown that residents are uniquely positioned as front-line workers to identify contextual challenges that may affect their SE and quality of care [22]. In addition, resident-led initiatives – interventions selected and implemented by residents – have been found to improve the work environment, enhance wellness, and decrease burnout [23, 24]. As such, medical education may contribute to residents’ SE by providing opportunities for them to address relevant contextual challenges during training.

Moreover, and without dismissing what residents go through, inevitable challenges may also provide opportunities for building resilience and adaptability [16, 25]. Effective self-regulating capacity helps people to manage adversity and to optimize their functioning [26]. Self-regulation involves “planning, generating, controlling, and adjusting, thoughts, feelings and actions in order to achieve personal goals and adapt to one’s changing environment” [26]. In relation to SE, self-regulation is argued to be a vital career skill that enables individuals to adapt to volatile work environments [1] and to attain value from work [27]. This mechanism may explain why self-regulation is positively associated with health, well-being, and work performance [1]. Research on residents’ self-regulating capacity corroborates these findings [26]. The capacity to self-regulate is initially learned from observing and imitating others [28–30] but can develop and change through awareness, instruction, practice, and support [26, 28]. As a result, medical education may be an important intervention period for developing functional self-regulation, likely benefiting residents’ SE throughout their careers.

Considering the importance of context and self-regulation for residents’ SE, it may be worthwhile to explore how medical education can contribute to residents’ SE. Medical education may foster residents’ SE by providing residents with the tools and opportunities to improve the contexts in which they perform and/or to teach residents how to self-regulate in a conscious and functional manner [28]. With that potential in mind, we observed how a pilot of an extracurricular educational program called the Resident Leadership Program (RLP) unfolded. The program’s main aim was for residents to develop leadership skills by implementing a local quality improvement project that contributed to residents’ SE in their local contexts. As such, we used this program to explore how medical education (here: the RLP) enabled residents to learn about, and to work on, their SE.

## Methods

### The resident leadership program

The RLP was a 6-month extracurricular leadership development program initiated by a collaboration of medical specialists and employees of the Dutch Health and Youth Care Inspectorate (the Inspectorate). The Inspectorate is an independent governmental agency tasked with regulating the quality of health and youth care in the Netherlands. It regulates health and youth services by ensuring compliance with laws and regulatory standards, promoting safe care, and stimulating health care providers to improve the quality of care. The Inspectorate was involved in the RLP because it considers the sustainable employability of health care workers an important theme for sustaining (future) quality of care in the context of ageing societies [31]. The first edition of the RLP —a pilot—started in February 2021 in a hospital in the North of the Netherlands. All residents in the hospital had been invited to apply for the program with a motivation letter. In the end, eight residents voluntarily applied, and they were all accepted to the program. The program took place online through Microsoft Teams due to COVID-19 restrictions at the time. The program consisted of five plenary course days, approximately once a month, with (guest) lectures on topics such as project management, change management, leadership, and sustainable employability. Additionally, participants were required to initiate quality improvement (QI) projects in smaller teams of two or three residents aimed at improving residents’ SE in their local contexts. Resident teams were assigned to a mentor physician. At the end of the program, the resident teams presented their QI project and findings at a symposium for several stakeholders, including hospital board directors.

### Context

This study was conducted in the Dutch hospital context, where residents who have graduated from medicine train in hospitals to become medical specialists, such as surgeons or gynaecologists. Residency takes between 4 and 6 years, depending on the specialty, and usually requires residents to work in both academic and non-academic hospitals.

### Study design

It is important to note that the RLP’s main aim diverged from this study’s main aim. Although we aimed to explore the RLP’s *potential* to enable residents to learn about and work on their SE, the RLP organizers aimed to develop leadership skills for residents through QI practice. In addition, as the RLP was an educational program, the RLP organizers were set on cultivating knowledge and skills pertaining to leadership and QI work, meaning that successful implementation of the QI projects was not a goal per se. These differences in aims mean that the RLP components were never based on our theoretical underpinnings. We took that limitation into account when formulating our research question, where we explicitly sought to explore, rather than evaluate, the potential of the RLP to contribute to residents’ SE as a secondary or unintended outcome.

In this study we took a qualitative approach because we reasoned that ‘learning’ how to sustain SE is very subjective, situated, and individually constructed, and hence, better grasped qualitatively [32]. To that end, we used different qualitative research methods to understand, capture and contextualize residents’ ‘learnings’ as a result of participating in the RLP. First, we interviewed residents (n = 8) individually in accordance with ‘in-depth interviewing’ prior to the start of the program [33]. These semistructured interviews served to establish a ‘baseline’ by eliciting residents’ preconceived ideas on sustainable employability, the context of medicine, and their lived experiences navigating through that context. Second, we engaged in participant observation [34] throughout residents’ participation in the RLP for 6 months. In that way we were able to experience first-hand how residents were introduced to sustainable employability, how they engaged with the concept in practice, but also to acquaint with the participants, organizers, and the program itself. Third, residents were interviewed again after the program, yet this time in groups (n = 3) corresponding to their project teams. Here, we invited residents to reflect together on what they had learned about (their) SE by participating in the program.

### Participants

Eight residents participated in the first edition of the RLP. Residents were specializing in gynaecology [2], pathology [1], surgery [4], and paediatrics [1]. The group consisted of three male and five female residents. Five residents were in their final year of residency, two were in their third year, and one had just started residency.

### Data collection

Residents participating in the RLP were approached by e-mail before the start of the program and were informed about the study aims, data collection methods, and intended usages of the data. To avoid participation bias, we specifically stressed that participation in the study was voluntary and not a mandatory part of participating in the RLP. In addition, we emphasized that we would treat data confidentially. Informed consent was obtained from all participants. The first author interviewed all eight participants individually prior to the start of the leadership program in February 2021. The interviews were semi-structured to allow for conversation to flow naturally yet within the confines of the research aims. The interview topics included residents’ understanding of SE, contextual factors important to SE, self-regulation, and ideas for intervention. On average, the interviews lasted 45 min. In addition, the first author observed meetings from the RLP organizers (9.5 h), plenary course days (30 h) and mentor team meetings (5.5 h) to understand how both the RLP and the resident team projects were unfolding from February to July 2021. Finally, three group interviews were held with each of the resident teams in January 2022 to reflect on the program. Group interviews had a more structured approach with clear interview questions to enable all groups to reflect similarly on their projects and participation in the RLP. The group interviews lasted approximately 70 min. Both individual and group interviews were conducted online via Microsoft Teams due to COVID-19 restrictions at the time and were audio-recorded and transcribed verbatim.

### Data analysis

The first author conducted the analysis (i.e. reading and coding interview transcripts) and held biweekly meetings with the other two authors to discuss and reflect on the data-analysis and preliminary findings The interviews and group interviews were coded in Atlas.Ti in accordance with ‘flexible-coding’ [35]. This approach, developed by Deterding and Waters (2018), takes advantage of qualitative data analysis software to organize, navigate and reduce qualitative data easier, making the process of coding, in contrast to open coding, less time consuming, tedious, and chaotic. The approach first stipulates that transcripts should be indexed by creating codes that represent the broad topics of the interviews, essentially connecting questions and answers throughout the data. This facilitates locating relevant data segments for analysis. In this study, those codes referred to the concepts we introduced in the introduction, such as ‘self-regulation’, ‘context of medicine’, and ‘sustainable employability’. Thereafter, the first author read through the transcripts and created memos for each document, with emerging ideas, relationships, and meaningful quotes [31]. After this step, the indexed codes were used to locate relevant data extracts, and to apply analytic codes.

As our aim was to understand how the RLP had enabled residents to learn about, and work on, their SE, we realized that it was essential to, first, contextualize our findings by sketching a status quo. That is, to contextualize residents’ learnings by first delineating how residents experienced and perceived their employment context, how it affected their SE, and how residents managed these contextual challenges through self-regulation. In that way, we could establish which residents’ learnings were attributable to the RLP. We present our findings in four ‘acts’ to mirror a play where cast (i.e., residents) and stage (i.e., context) may change as a result of unfolding events (i.e., RLP). It is important to note here that the findings of this study are based on a set sample size of eight residents, and as such, we cannot claim theoretical data saturation.

### Reflexivity

IvdV was the primary researcher and conducted this study as part of her PhD trajectory on the SE of health care workers. She has two master’s degrees, one in organizational learning and development and one in strategic occupational health. IvdV was supervised by JWW, an associate professor in quality, safety, and professional work in health care, and IL, trained as a physician, but currently working as a professor in health care regulation. Our various theoretical and professional backgrounds enabled us to collect and analyse the data in a holistic manner. In addition, all authors have ample knowledge and experience with conducting qualitative research in health care settings, albeit from different disciplines, which enabled us to explore the themes in this research in a balanced manner. Moreover, our previous research on the SE of practicing physicians also inspired us to more explicitly focus on the role of context and self-regulation to understand residents’ SE. Finally, none of the authors had any prior connection to the study participants.

## Results

### The prologue

We present our findings in four ‘acts’ mirroring an unfolding play as a metaphor to show how residents’ understanding of context, self-regulations, and quality improvement work—relevant to their SE—changed in the RLP. The first two acts provide necessary context information to understand the findings in the latter two acts, where the research question is answered. The first act, *setting the stage*, sketches how residents perceived the context of medicine and how it challenged their SE. The second act, *acting the part*, demonstrates how residents, prior to participation in the leadership program, navigated that context and used self-regulations to sustain their SE. The third act, *changing the décor*, shows how residents tried to change their local employment contexts through QI projects aimed at improving residents’ SE. The fourth and final act, *‘growing one’s role’*, describes the reflections and learnings of residents by participating in the program that are relevant to their SE. Table 1 provides an overview of data sources, acts, codes, and accompanying quotes.

**Table 1:** Overview of results in ‘acts’, including data sources, codes, and example quotes

Data	Act	Explanation	Code plus example
Individual interviews (*n* = 8) conducted prior to residents’ participation in the RLP	1. Setting the stage	Participants sketched their perception of the context of medicine and how it relates to their SE	Context: high expectations and standards: *“Much is expected of you*,* such as that you continue work after scheduled hours*,* which already amounts to 46 or 48 hours plus over time. You also see that it is no longer enough to excel clinically to be considered a good doctor*,* but it is also expected that you engage in health care developments*,* include patients etc. And do not get me wrong*,* those are good developments*,* medicine is much more than clinical reasoning. (…)*,* but it does add to the pressure. On top of that*,* you see more and more women working in health care*,* that also have families they want to spend time with*,* have a social life*,* want to have hobbies. So*,* the bar is set quite high by others*,* but also us (…). I do not think that this current way of working*,* or the way we organize our profession*,* is sustainable for careers spanning forty years.”* (resp. 5)
	2. Acting the part	Participants explained how they managed contextual challenges through self-regulation	Self-regulation: accepting that if you want something it may require sacrifice: *“As a resident [not in training]*,* I once realized that you know when you are at the right place. And at [specialty]*,* I am in the right place. That made me realize that*,* where or in what way*,* I will be accepted to a [specialty] residency. And you need to be prepared to sacrifice things*,* such as having to travel all over the country. And I try to let that go and tackle each move when it comes*,* even though it is uncomfortable for my family (…).”* (resp. 2)
Participant observation throughout the RLP Group interviews with every resident team (*n* = 3) after the RLP	3. Changing the décor	Participants described their quality improvement project aimed at improving residents’ SE in their local contexts. Participants reflected on the rationale of and the reality behind implementing their quality improvement projects.	Description project 2: “*Our project is called ‘residents in the lead’. So*,* we have developed a vacancy database for committees in the hospital that residents can apply for. (…). So*,* we have approached committees and every now and then we approach residents*,* and if they are suited*,* we introduce them to the committees*,* and the latter approve or not*, * and then we match them.”* (team 2, resp. 2) Rationale SE project 2: “*Well*,* if residents get the impression or can actually have a say in matters that concern them or how things are organized in the hospital*,* it will make them feel better. It would make them feel more involved*,* heard and above all it allows them to demonstrate their professional development and leadership skills.”* (team 2, resp. 2) Implementation project 2: *“(…) the goal was also to embed this project into the hospital’s structure*,* so it would not depend on our efforts. It needed to become something ‘normal’. So*,* we involved the Academy*,* so they could instruct new residents during onboarding days about the option to join committees as a resident.”* (team 2, resp. 3)
	4. Growing one’s role	Participants explain what they learned from participating in the RLP that was relevant to their SE	Lessons learned: awareness of importance SE: *“Resp. 1: More awareness*,* I think. Resp. 2: Certainly. At first the concept of sustainable employability was a bit vague. I’d heard about it before*,* but I never realized what it entailed. (…). I know for myself what I need to be mindful of now. Whether that is eating healthy*,* not doing anything for half an hour*,* to state a few examples. I will certainly take that with me and keep that in mind. Absolutely.”* (team 1)

### Act 1: setting the stage – how residents experienced the context of medicine

In this part of the results, residents sketched the context in which they currently or previously worked. Residents mainly elaborated on contextual factors that they perceived as challenging or problematic to their SE. We divide these challenges into organizational, team-related, and professional contextual challenges.

Organizationally, residents expressed that their educational needs and requirements often conflicted with, or were subordinated to, patient care. One of the interviewees summarized this as follows:

*“A lot of decisions*,* from directors*,* management*,* specialists*,* are made based on whether there are enough ‘pawns’ or whether shifts are covered. (…) But*,* when it comes to looking what that person needs in their education or to discuss what would be a suitable internship*,* that only receives attention temporarily. Also*,* if these needs or wishes in any way affect shifts (…) we become ‘pawns’ again.”* (resp. 1).

On paper, residents explained that they are supposed to be superfluous employees and that 10 h of their 48-hour contracts are unpaid and dedicated to their training. In practice, however, they are “indispensable” to patient care and hence deployed to handle patient consults and ward rounds, often including those 10 h (resp. 7). According to residents, scheduled hours for patient care did not cover the vast amount of work to be done. As a result, residents regularly performed these tasks (e.g., administration, preparation) outside of work hours, usually without appropriate compensation. It also meant that time for specialty-relevant clinical encounters and time to study largely took place outside of regular work hours. Residents reported that this resulted in stress, fatigue, diminished joy at work, and decreased time to recover from work. One of the residents explained:

*“At the end of the day around 5 or 6 PM*,* if you are ready with obligations*,* you start on the departments’ administration*,* squeeze in a few family conversations*,* and then yes*,* you walk out of the hospital at 7:30 PM*,* home by 8:30 PM. And then I still need to eat*,* work out*,* and maintain a good relationship.”* (resp. 4).

Regarding organizational human resource services, most mentioned there to be no specific (known) policy or interventions that concerned residents’ SE. A few, however, referred to the ‘Fit-to-Perform’ program, which is a self-assessment intervention and campaign to prevent sleep-deprived surgeons from going into surgery. Nevertheless, most residents emphasized that basic, yet fundamental, employment conditions enabling undistracted functioning at work were not well arranged or not even offered. Examples included travel arrangements, personnel substitution, or pensions. One resident expressed frustration about that:

Resp. 1: *“Look*,* it is kind of arranged in the following manner: there is a shift pool with a number of shifts that need to be covered*,* period. However*,* if people get sick or are on leave*,* or something like that*,* the others must then simply fix that amongst themselves*,* without support. That is kind of a weird concept*,* right? Ridiculous even. Normally*,* if someone gets pregnant or cannot cover a shift for some time*,* someone else should show up”.* Interviewer: ***“****You mean*,* as in an extra person”?* Resp. 1: *“Exactly. That should be a normal phenomenon. In every branch it is considered normal to hire someone*,* temporarily. But here it is consistently not arranged”.* (resp. 1)

Regarding team-related challenges, residents mentioned a lack of support in groups to develop themselves into physicians who mirror their personalities, interests, and talents. In addition, residents noted that the atmosphere at work is often tainted with slumbering conflicts. They also experienced rather impersonal contact with colleagues at work, where colleagues seemed reluctant or unwilling to invest in building personal connections at work. This resulted in awkward situations, as one resident explained:

*“There is a lack of interest in getting to know the person behind the resident. For example*,* when you give birth*,* you return to work*,* and only on the first day people inquire how you are. After that*,* it’s done*,* and nobody asks anything*,* even though you spend hours and hours with the same colleagues and despite me being interested in them”.* (resp. 2)

Residents explained that this was a missed opportunity, as knowing your colleagues better is likely to create goodwill, which in turn helps colleagues to sympathise with you or even cut you some slack in the event of personal circumstances.

Professionally, residents felt that they were not (being) sufficiently prepared for increased job requirements as a physician, such as being able to conduct quality improvement work and ensuring patient empowerment. As one resident summarized, you are being trained to become a physician *in theory* (i.e., clinical knowledge/skills) but not what it entails to work as a physician in *practice* (i.e., contextual knowledge/skills). Residents also explained that they were not very informed, involved or consulted in organizational decisions made at the department or hospital level.

### Act 2: acting the part – how residents self-regulated their SE

In this section, we present the self-regulations residents enacted to sustain their SE in their local employment contexts. Self-regulations are categorized into residents regulating themselves, regulating work, regulating others and regulations that were not perceived as possible.

First, residents regulated themselves. In the absence of organizational conditions that facilitated functioning well, residents created “small victories” themselves, usually by circumventing the “official route” (resp. 1). One example included arranging a nearby parking space to significantly limit travel time. Residents also gradually waived initial system-level frustrations or job insecurities that hampered their SE and replaced these with a form of acceptance to “avoid becoming insane” or to sustain joy in work (resp. 2). Similarly, some had accepted consistent work-life imbalance as a sacrifice for better career opportunities after residency. In addition, many residents consciously and actively sought to broaden their employability by investing in professional development. They engaged in extracurricular courses, such as the RLP, took up extra roles at work, and occupied themselves with research and teaching. One resident mentioned consciously planning moments for reflection:

*“Once every now and then I plan a reflection moment with myself. That can be anything. Walking*,* writing in a diary (…)*,* but it needs to be a conscious exercise. (…). I consciously reflect on questions such as ‘why do I like this?’. ‘Why do I do this’? ‘Is this still what I want’? (…). ‘Are there things I’d like to do differently’? And as long as the conclusion to myself is ‘this is what I want’*,* then I think everything is alright”.* (resp. 5)

Second, residents regulated work. One resident regulated workdays and started working part-time (i.e., working four days) to maintain time for other activities, such as research and family. While it did prolong residency by 1 year, this resident also argued that it provided “more personal space” and guaranteed longer employment in an insecure job market (resp. 6). Another resident had regulated late work hours on certain evenings by planning sport activities that were labelled “sacred”, which could not be cancelled due to work (resp. 4). Furthermore, one resident regulated his work schedule by switching medical specialties. The other specialty and the way work was organized offered more control over his work schedule, which was perceived to be a better fit. The following quote further illustrates that resident’s decision:

*“I was thinking about my sustainable employability (…)*,* as I had quit that other residency program for several reasons. (…) [and] if I had continued with that specialty*,* I would not have lasted another 30 years. So*,* in terms of sustaining my employability*,* I must have realized early on that the first residency was not sustainable. Knowing what I did not want also helped me to pick the right residency program”.* (resp. 3)

Third, residents’ regulated others. Often from the motivation that they had missed certain interactions themselves. Some consciously sought to be a good example to others, and others considered it important to show a genuine interest in the personal lives of peers. Someone else deliberately provided positive and constructive feedback to colleagues. One resident felt the responsibility to guard the interests of other, often younger, residents:

*“Sustainable employability is a very big theme. Personally*,* I could either work on that myself*,* or possibly the people close to me (…). Here*,* I am not the oldest resident*,* but in previous hospitals*,* I often was. In that case*,* you are the ‘watchdog’ for other residents to make sure that they are not allocated too many shifts or that the same one’s always stand up to supervisors”* (resp. 1).

Fourth, residents also elaborated on self-regulations they wished they could enact but were impeded in some way. For example, a few residents mentioned that the hospital had not allowed them to document their overtime hours to demonstrate, and in turn limit, structural overtime. One resident explained that if all residents in the Netherlands would document their overtime, “all [Dutch] hospitals would go bankrupt within two months” (resp. 1). In addition, some residents did not always feel comfortable seeking help or voicing concerns to their supervisors. They perceived themselves to be too dependent on their supervisors’ performance assessments, which were considered relevant for future career prospects. Finally, one resident mentioned that taking care of patients is so engrained in their sense of duty that taking care of themselves is often overlooked:

*“We have to take care of patients*,* but actually also ourselves. Yet*,* we are always the ones that continue working the longest*,* even though we have a fever. As long as we can stand on our legs*,* and don’t fall*,* we continue.”* (resp. 5)

### Act 3: changing the décor – how residents opted to change their local contexts

#### Plans

As part of the RLP, residents designed and implemented QI projects in teams aimed at improving residents’ SE in their local contexts. A description of the content and rationale behind these projects provides a glimpse of what residents perceive as important for their SE and how, when ‘in the lead’, they intend to achieve change.

The first resident team aimed to facilitate detachment from work by enabling undisturbed breaks through a system of pager-handover. They argued that regular detachment from work relates to SE because it can sustain joy in work, invigorate residents for the remainder of their shifts, and increase productivity. This, in turn, could prevent burnout, sickness absence, and working overtime. The second resident team aspired to facilitate resident participation in hospital committees by acting as an intermediary that matches interested residents with interested committees. This would favour SE, because participation enables residents to influence relevant decision-making processes and provides them with opportunities to develop leadership skills. The third team aimed to develop an assessment for residents as input for a personal development plan. This team asserted that residents who obtain insights into their talents and interests would make more informed decisions on suitable career paths and activities. This would increase their job satisfaction and broaden their employability beyond their clinical specializations. For an overview of the projects and additional information, see Table 2.

**Table 2:** Description of residents’ QI projects, including teams’ rationale for residents’ SE and quality of care, and their experiences with implementation

QI projects	Description projects	Relationship to residents’ SE	Relationship to quality of care	Implementation in practice
**1.** **Detachment from Work**	Facilitating detachment from work where residents can take undisturbed breaks to recharge in their own ways, by creating a system where residents temporarily take over each other’s pagers whilst detaching, so patient care can continue.	Regular detachment from work sustains residents’ joy in work, invigorates residents for the rest of the shift, and improves productivity. This, in turn, prevents burn-out, sickness absence and working overtime.	Taking breaks leads to healthier doctors, decreased medical errors, and more friendly and emphatic interaction with patients.	Project was implemented in two departments. Difficulties to implement properly included staff shortage (i.e. temporarily increasing staff’s workload when taking over pagers for other staff’s breaks was considered undesirable/unfeasible); lack of space to facilitate undisturbed detachment; a culture where residents were not used to or felt uneasy to take breaks or considered to not need any breaks.
**2.** **Participation in Hospital Committees**	Facilitating opportunities for residents to participate in hospital committees, by seeking and convincing committees to participate and subsequently, matching them with interested residents.	Participation in committees enables residents to be involved in decision-making processes in the hospital that may affect their own SE. Opportunity to participate also signals to residents that their perspective is heard and taken seriously. Finally, participation allows residents to demonstrate and develop leadership skills.	Residents with first-hand and front-line experience possess valuable information to inform (quality/safety) issues in these committees.	This project was successfully and sustainably implemented. Seven residents were matched with seven different committees. Reasons for successful implementation included: a concrete and cheap project requiring little effort from committees; actively name-dropping important local stakeholders that supported the project; collaborating with hospital actors that shared a mutual goal (e.g., L&D).
**3.** **Personal Development Assessment**	Creating an assessment for residents in the hospital as input for a personal development plan.	An assessment provides residents with insights into their talents and interests, in turn, enabling them to make more informed decisions on career paths, likely to increase job satisfaction. It also allows residents to develop themselves more broadly, also increasing their employability. An assessment facilitated by the hospital during work hours also prevents residents to work on personal development outside of regular workhours, improving work-life balance.	Happy doctors with limited job insecurity are likely to provide better, undistracted care. Acquired self-knowledge also helps hospitals to employ ‘the right person at the right place’, benefiting quality of care.	The team managed to find an external party that could customize a digital assessment for residents at the hospital. Yet the project proved hard to be approved by the hospital as more parties got involved, increasing the scope, and in turn, the financial ramifications.

### In practice

The first resident team, which facilitated detachment from work, implemented their project in two different departments and learned that implementation in practice was complex. Departments experiencing a shortage of staff considered it undesirable or unfeasible to temporarily increase the workload of staff by taking over each other’s pagers when others were detaching. There was also a lack of space to facilitate undisturbed detachment from work. The team also encountered a persistent culture where despite facilitating detachment, residents still felt uneasy to take breaks, perceived to not need any breaks, or were not accustomed to taking breaks. Finally, the team concluded that their argument on how detachment could improve productivity and prevent working overtime did not hold up in practice, as illustrated through the following quote:

Resp. 2, team 1: *“I can drop off my pager for half an hour*,* quickly put on jogging pants*,* and run for half an hour and return*,* but the rest of the organisation will have continued and not everyone took a break*,* naturally. So*,* I do not think that leaving for half an hour will result in a return on investment in productivity (…). There are a thousand pieces to the puzzle that all have a part the play in the hospital. If that radiology test has not been executed*,* then the anaesthetist can’t come for a pre-operative conversation”.* Interviewer: *“I see. So*,* there is still a situation where others need you*,* and you need others*,* and that does not necessarily become less if you are more productive due to having detached”?* Resp. 2, team 1: *“It is all or nothing. Everyone would need to participate.”*

The second resident team, on residents’ participation in hospital committees, was the only project that was implemented within the 6-month program that continued afterwards. As a result of the teams’ efforts, seven residents in the hospital were matched with seven different committees by the end of the RLP program. The team attributed several reasons to the successful implementation of their project. First, the project was very concrete, did not cost the hospital any money, and would require limited effort from committees. Second, the team actively name-dropped important local stakeholders who supported the project idea when meeting and convincing committees to collaborate on the project. Third, the group collaborated with specific parties within the hospital that shared a mutual interest in this project, which, in turn, advanced implementation. For example, the team arranged for the resident’s association to support and promote the program and for the in-house academy to create a structure for the matching process.

The third resident team aimed to create an assessment for residents’ personal development. The team managed to find an external party that could customize a digital assessment for residents at the hospital, but it proved difficult to implement in the hospital for several reasons. Most importantly, the team acknowledged how they got carried away as more parties, both internal and external, became involved in the project, making the project more extensive and difficult to implement. In addition, the financial ramifications also increased as the scope increased, which made it more difficult to obtain project approval.

### Act 4: growing one’s role – what residents learned from participating in the RLP

In this section, we report on residents’ reflections on what they have learned from participating in the RLP that is relevant to their SE. First, residents explained how they had grown more conscious of the meaning and importance of SE and sometimes adjusted their self-regulation in ways that would benefit their SE. One resident explained having dared to address educational needs because of the program, whereas prior this was deemed difficult:

*“For my future employability*,* I have realized that if you want something badly*,* there is actually quite a lot of opportunity to realize it*,* even during residency and despite that there is no formal opportunity. It is possible to arrange for things in residency. [Gives example specific to specialty]. That relates to the RLP*,* because even though there is no formal time dedicated to this [example] activity*,* I still let my supervisor know that I wanted this badly. So yes*,* in a sense*,* I have learned that if you want something*,* you just need to address it to the right person*,* and then it is obtainable.”* (resp. 1, team 1).

Another resident had become more aware of the importance of taking proper care of oneself to sustain SE through exercise, diet, and relaxation. Finally, another resident explained having set boundaries on working overtime when applying for a new job.

*“I was applying for a new job whilst participating in the RLP. At [location] there was an offer*,* but I also knew that there was a lot of work pressure*,* which also came up during my job interview. There*,* they asked me how I was going to manage that. I stated clearly that I would not shy away from working hard*,* but that I also did not intend to burn myself out. Apparently*,* they agreed*,* because I got hired. So*,* those are things I am conscious of now. In addition*,* I also make sure I go home at a decent time as well.”* (resp. 3, team 3).

Second, residents developed more institutional and organizational sensitivity, which helped them understand *and* navigate the context of medicine more easily and strategically. By practicing QI work, residents learned about the importance of networking and ensuring ‘buy-in’ from the right actors inside the hospital. They also learned to leverage hospital actors’ interests, experience, and capabilities to advance the implementation of their projects. In addition, residents’ contextual sensitivity also expanded beyond the walls of the hospital. The involvement of the Inspectorate in the RLP enabled residents to become acquainted with the Inspectorate’s work and their take on the sustainable employability of health care workers. Some residents explained that the involvement of the Inspectorate in the RLP had made them change their—often negative—perception of the Inspectorate:

Resp. 3, team 2: *“I think that the image of the Inspectorate is not extremely positive. If anything*,* you are taught to be wary of them: ‘Best make sure to organize your administration*,* or else the Inspectorate might sue you’. (…). It would be good for residents to be shown the positive sides of the Inspectorate*,* which we have seen in the RLP”.* Interviewer: *“Which is”?* Resp. 3, team 2: *“That they are not just some kind of watchdog that gives a rap on the knuckles*,* but that they actually try to make health care better (…).* Interviewer: *“What exactly made you change your image”?* Resp. 3, team 2: *“Just*,* the way in which they explained themselves*,* how they conversed*,* (…) and that they do really look with a humane perspective (…).* Resp. 2, team 2: *“And that they are open to many improvements and that they want to support bottom-up initiatives. They now seem more of a sounding board (…)*,* instead of an institution that imposes”.*

Furthermore, practicing with the different steps and tenets of QI work allowed residents to acquire more understanding of a layered and complex hospital context and was deemed useful for other work-related activities.

Resp. 1, team 2: *“I thought the theory on how to set up a project was interesting*,* even if it sounds logical. As soon as you actively engage with the steps of QI work*,* you notice that it is helpful*,* and I still use and apply that same thinking. (…) [For example]*,* we are short staffed now and we are trying to fix that. But now I do that in a more structured manner*,* talking to the right people*,* making everything more concrete*,* (…) thinking what is the next step? Deciding on a plan of action*,* trying to get feedback on it*,* setting deadlines and dissecting everything into smaller pieces****”.*** Resp. 2, team 2: *“(…) I am also trying to set something up in my department. The steps that we learned I take with me. I no longer start with a blank canvas”.*

Third, participating in the program and initiating QI projects in practice gave residents a new sense of purpose and fulfilment. Residents felt useful to their peers and experienced joy at work when being enabled to initiate projects that contributed to peers’ SE. Thus, by helping other residents’ SE, residents also indirectly worked on their own SE.

Resp. 3, team 1: *“It might seem silly*,* but when you have worked in patient care for a long time*,* and experienced the same old things*,* such as seeing the same twenty patients every Friday*,* well*,* then at a certain point you no longer have that feeling (…) of adding value to other people’s lives. (…) and that is why it is so fun to set up these projects (…)”.* Interviewer: *“You mean that you feel fulfilled*,* maybe not by helping patients*,* but in this case*,* helping your colleagues”?* Resp. 2, team 1: *“Yes*,* yes*,* yes*,* exactly! (…) that you do not only do what you are being told to do*,* but actually actively…****”.*** Resp. 1, team 1: ***“****…contribute to something for the long run”.*

Finally, although engaging in QI work was first and foremost intended to be a learning experience for residents by the RLP organizers, the resident teams did reflect on the successes or sustainability of their projects. One group, whose project did not continue after the RLP, mentioned that it would be nice to learn how to strike a balance between having seemingly unlimited scope and managing the feasibility of the projects within a limited time frame.

### Epilogue

In sum, Act 1 showed that residents experienced multiple challenges in their local employment contexts that affected their SE. Act 2 showed that residents dealt with these extensive challenges through an equally elaborate array of self-regulations. Act 3 demonstrated that residents seemed capable of identifying, addressing, and sometimes even changing relevant contextual issues when they were taught about and enabled to practice QI work. Act 4 concluded that by engaging with the concept of SE through QI practice, residents have increased their awareness, understanding and perceived importance of SE, which for some residents was reflected in their adjusted self-regulations. Moreover, QI practice enabled residents to develop important skills that will likely benefit their SE in the future, such as institutional and organizational sensitivity and the ability to interact with a multitude of actors in a multi-layered health care context. And finally, residents enjoyed themselves and felt useful when contributing to their peers’ SE through QI work. Thus, QI practice on the topic of SE seemed to have fostered residents’ self-regulating capacity, both in terms of knowing how to regulate themselves and regulating their environment more effectively.

## Discussion

Medical education may contribute to residents’ SE by providing opportunities and tools for residents to (I) improve the contexts in which they perform and (II) develop functional self-regulation. To that end, this study explored the potential of the Resident Leadership Program (RLP), an extracurricular leadership development program, to enable residents to learn about, and work on, their SE.

This paper confirms that the Resident Leadership Program enabled residents to learn about, and work on, their SE in three ways. First, residents became more aware of the importance of their SE, and some consciously adjusted their self-regulation accordingly. This corresponds with research that advocates employers and governments to intervene as early as possible in younger employees’ SE by installing “early employability awareness” [36]. In addition, residents may have adjusted their self-regulation in the RLP as a result of activating self-regulating subprocesses that are considered preconditions for adjustment [37]. These processes constitute *self-observation* (i.e., what am I currently doing? ), *self-evaluation* (i.e., does it meet a goal or standard? ), and *self-reactive influence* (i.e., how can I attain this goal/standard? ) [37].

Second, residents felt more equipped to navigate and employ the contexts in which they performed. This suggests that residents have essentially been taught and enabled to better self-regulate their employment contexts. This aligns with previous research on how attending physicians who have completed residency manage to sustain their employability throughout their careers. Here, physicians indicated to use and need three types of self-regulation, of which ‘influencing work directly’ (e.g., “addressing dysfunctional work practices”) was one of those categories [38].

Third, residents felt more valuable to their peers and experienced joy at work when being enabled to initiate QI projects that contributed to their peers’ SE. Thus, residents indirectly improved their own SE when working on their peers’ SE. The importance of sustaining or reinstating residents’ joy at work has been advocated by many healthcare stakeholders as a means to prevent burn-out and to maintain quality and safety [39].

### Strengths and limitations

We consider our approach, i.e., our theoretical perspective and methodology, as a strength because it was able to uncover outcomes of the RLP that would have otherwise remained ‘hidden’. A limitation of this study is the specific Dutch context in which participating residents work, which may be different from the employment contexts of residents in other countries. However, it seems that residents, irrespective of context or educational system, share several similar challenges, such as workforce shortages, high burn-out rates, and unprofessional behaviours at work [40–42]. As such, we would expect that the mechanisms underlying our findings may still be of use, or may resonate, in different international settings. In addition, the findings from this study were derived from a relatively small sample that also volunteered to participate, and as such cannot be generalized to all residents. In a similar vein, because of the set sample size, we could not establish theoretical data saturation. Furthermore, although theory advocates for effective self-regulation to advance SE, we cannot conclude that residents’ adjusted self-regulation sustains in practice. Nonetheless, their narratives give us a hunch as to whether self-regulation can be cultivated and at what contextual challenges these are targeted.

### Implications for practice and research

The findings allude that being introduced to and learning about the concept of ‘sustainable employability’ in the RLP influenced how residents subsequently perceived and continued to ‘work on’ their SE. Whether this is due to its formal introduction in one of the lectures, in practice through quality improvement work, or by being interviewed about it remains to be examined in future research. In particular, we emphasize the need for longitudinal research that follow residents throughout their careers to understand how SE fluctuates, develops and which aspects of employment or training have a negative or positive effect on it. Nonetheless, it is likely that the theme that governed the first edition of the RLP influenced residents’ own SE via self-regulation. This suggests that building self-regulation competence is both a feasible and desirable educational outcome [43]. Moreover, we concur with Brydges and Butler [28] that self-regulation, although these are and remain self-directed practices, should still “be guided and supported as a form of co-regulated practice (i.e. practice that is shaped by context and by others)”.

Moreover, by instructing residents to engage in QI work that contributes to other residents’ SE, it appears that residents contribute to both their peers and their own SE. Therefore, we recommend that supervisors, educators, and/or managers involved in the medical education of residents capitalize on this win‒win situation by facilitating residents’ participation and engagement in QI work aimed at improving the work environment for those residents that are interested, or expected, to engage in QI work now or in the future [44–46]. This contrasts with most QI work, where there is a tendency to forget or ignore the implications of QI projects for clinicians’ workload, unintentionally aggravating their already challenged SE [47]. The residents in this study showed to be well situated and capable of identifying contextual challenges to their SE and addressing these challenges in straightforward and feasible ways, which is in line with the findings of other studies [48]. The RLP could serve as an example for educational practitioners to integrate educational requirements that stipulate the development of physician leaders proficient in quality improvement [48] *with* the pressing need to involve residents in improving the debilitating contexts in which they function. To avoid frustration, educational practitioners are advised to pay attention to the feasibility or sustainability of residents’ QI projects in order to safeguard residents’ interest for both valuable learning experiences and their desire to contribute successfully or meaningfully with their projects.

In addition, there is this premise in SE literature that SE should benefit both organizations (or society) *and* individuals, resulting in a shared responsibility to sustain employees’ employability [36, 49]. However, the tensions that arise from arguably very different interests around SE in practice (e.g. feeling fulfilled versus averting recruitment costs) are not often laid bare. For example, one of the residents in this study recalls a change in specialism, which due to its better fit was argued to be beneficial for his SE. His former employer, however, may have marked this as an undesirable organizational outcome, due to the budgetary and personnel consequences of his leave. Thus, despite a common interest on paper, conflicting or different interests in practice may hamper employers to wholeheartedly invest in individuals’ SE. Researchers are recommended to consider and reveal these tensions, and policy makers and regulators should be more sensitive to these different interests when aiming to contribute to the SE of individuals.

To conclude, the findings from this study seem promising given the prevalence of resident impairment *and* the resulting need to develop resilient residents who can reflect and act upon their SE in systems that are likely to remain unsettling. This is not to suggest that there is no (system) improvement possible [50], nor does this imply that residents should be made solely responsible for sustaining SE [16]. Instead, it suggests that it is important, and possible, to equip residents with the tools, opportunities, and support to sustain their SE in medical education, which will likely serve both the residents and their patients in the rest of their careers.

## Data Availability

The datasets generated and/or analysed during the current study are not publicly available because of the privacy of the study participants, but the data supporting the analysis (codes and underlying (anonymized) quotes) are available from the corresponding author on reasonable request.

## Abbreviations

SE: Sustainable Employability
RLP: Resident Leadership Program
QI: Quality Improvement
